# Effect of Bowel Preparation to Colonoscopy Interval on Preparation Quality and Colonoscopy Outcomes: A Meta-Analysis

**DOI:** 10.5152/tjg.2022.22033

**Published:** 2023-01-01

**Authors:** Ying Gao, Xi-jie Lin

**Affiliations:** 1 Department of Gastroenterology, Heilongjiang Provincial Hospital, Harbin, Heilongjiang, China

**Keywords:** Bowel, cleansing, colonoscopy, interval, preparation, time

## Abstract

**Background:**

This study evaluates the effect of bowel preparation to colonoscopy time interval on quality of bowel preparation and outcomes of colonoscopy.

**Methods:**

Studies were identified after a literature search in electronic databases and were selected for inclusion based on precise eligibility criteria. Meta-analyses of proportions were performed to achieve overall bowel preparation adequacy and adenoma/polyp detection rates. Odds ratios depicting associations between bowel preparation quality and bowel preparation to colonoscopy time were pooled to achieve an overall estimate.

**Results:**

Twenty studies (10 341 individuals subjected to colonoscopy) were included. Bowel preparation adequacy rate was higher with shorter (94% [95% CI: 91, 97]) than with longer (84% [95% CI: 79, 89]) interval between bowel preparation and colonoscopy. In a subgroup analysis, <5, 6-10, 11-20, and >20 hours intervals were associated with 94% [95% CI: 92, 97], 92% [95% CI: 86, 96], 85% [95% CI: 77, 91], and 85% [95% CI: 75, 92] adequacy rates, respectively. A pooled analysis of odds ratios also showed that bowel preparations adequacy was significantly better with shorter bowel preparation to colonoscopy time (odds ratio 1.69 [95% CI: 1.23, 2.15]). There was no significant difference in adenoma detection rate between shorter (18% [95% CI: 9, 29]) and longer (19% [95% CI: 15, 22]) bowel preparation to colonoscopy intervals. Polyp detection rate was higher with shorter (47% [95% CI: 27, 68]) than with longer (30% [95% CI: 24, 38]) bowel preparation to colonoscopy interval.

**Conclusion:**

A shorter interval between bowel preparation and colonoscopy led to a higher bowel preparation adequacy rate which was also associated with a higher polyp detection rate.

**Main Points**

- Bowel preparation to colonoscopy time affects the outcomes of colonoscopy.
- A shorter interval between bowel preparation and colonoscopy leads to higher bowel preparation adequacy rate.
- A shorter interval between bowel preparation and colonoscopy may yield a higher polyp detection rate.

## Introduction

Colorectal cancer (CRC) is the third leading cause of cancer-related mortality.^1^ In the United States, by January 2019, 776 120 men and 768 650 women had CRC or a history of CRC. Approximately half of this population was over 65 years of age. The estimated number of CRC cases in the United States in 2020 was 147 950, and 53 200 individuals died due to CRC this year. The incidence rate per 100 000 individuals increases steadily from 10.5 in the 35-39 age group to 259 in the 85+ age group.^2^ The global incidence of CRC in 2020 was estimated to be 1.9 million cases, and 0.9 million deaths were attributed to CRC this year. The incidence was higher in men (23.4/100 000) than in women (16.2/100 000).^3^

Approximately 60%-70% of individuals are diagnosed at a middle or advanced stage of CRC which leads to higher mortality in comparison with those who achieve early detection and treatment.^4^ Screening tests can prevent disease and mortality and reduce the healthcare costs which are associated with better quality of life. Screening for CRC has led to decreased mortality in the last 2 decades.^5,6^ The CRC develops in a stage-wise manner where benign outgrowths called polyps gradually turn into tumors and metastases. Such changes are accompanied by the histomorphological and genetic/epigenetic changes which develop over time; therefore, earlier detection of a pathological change in the colon or rectum can prevent CRC or improve prognosis.^7^

The CRC screening with high sensitivity fecal occult blood test every third year, sigmoidoscopy every fifth year, or colonoscopy every 10 years is recommended for individuals over 50 years of age with an average risk of CRC.^8^ Such screening strategies are found to reduce CRC mortality, and different screening methods are found to provide comparable survival benefits.^5,6^ Colonoscopy is not only a screening tool but is also required for the treatment and surveillance of colorectal lesions.^9^ It has been reported that the use of colonoscopy for removing adenomatous polyps can reduce mortality by up to 53%.^10^ Besides its use for primary screening, colonoscopy is also used to verify the outcomes of other screening techniques such as the fecal occult blood test.^11^

Several factors such as the institutional cadre, personnel, hospital stay length, patient characteristics, and bowel preparation efficiency can affect the outcomes of colonoscopy.^12^ Adequate bowel preparation is one of the most important requirements for achieving high-quality colonoscopic findings. Many studies have reported that a considerably higher proportion of patients undergoing colonoscopy have inadequate bowel preparation.^13^ Within 5 years, approximately 2 in 1000 individuals develop CRC due to lesions missed by the baseline colonoscopy.^14^

Two main schedules for bowel preparation are the split and same-day preparations. A meta-analysis has found that both split and same-day bowel preparation methods provide comparable outcomes, although split-dosing leads to better compliance.^15^ However, many studies have reported that time intervals between bowel preparation and colonoscopy influence the outcomes of the colonoscopy. The objective of the present study was to conduct a systematic search of relevant studies and to perform meta-analyses of statistical indices indicative of the effect of bowel preparation to colonoscopy interval on the quality of bowel preparation and the outcomes of colonoscopy.

## Materials and Methods

This review included studies that (a) recruited patients undergoing colonoscopy and evaluated the effect of the time interval between bowel preparation and colonoscopy; (b) reported the outcomes with regards to the preparation quality in patients with shorter versus longer intervals between bowel preparation and colonoscopy; and (c) reported the outcomes of colonoscopy in patients with shorter versus longer interval between bowel preparation and colonoscopy.

Exclusion criteria were: (a) studies that focused on comparing split-dose with single-dose bowel preparation regimen; b) studies that involved the analysis of patients with poor bowel preparation only; (c) studies that evaluated colonoscopy delay time but not bowel preparation to colonoscopy time; (d) studies that reported the outcomes of colonoscopies performed at different times for individuals whom bowel preparation timings were similar or were not reported; (e) studies that evaluated the efficacy of reminders to patients for bowel preparation schedules; and (f) articles with qualitative information.

The literature search was conducted in several electronic databases including Google Scholar, Ovid, PubMed, Science Direct, and Springer. Relevant keywords were used as phrases. These keywords included colonoscopy, screening, endoscopy, colon, rectum, colorectal, bowel, preparation, cleansing, time, interval, duration, morning, evening, afternoon, runway time, quality, adequate, adenoma detection rate, and schedule. After identifying the relevant articles, bibliographic sections of important research and review articles were also screened for additional studies. The literature search encompassed research articles published during the date of database inception and November 2021. 

### Statistical Analysis

Demographic, clinical, and pathological data, study design, conduct, and analysis information, bowel preparation regimens and schedules, bowel preparation quality assessment scale scores, polyp/adenoma detection rates, bowel preparations ratings, and related statistical data were extracted from the research articles of the included studies. Quality assessment of the included studies was performed with the Cochrane Quality Assessment Tool for randomized controlled trial for randomized studies or with the National Institutes of Health Quality Assessment Tool for observational cohort and cross-sectional studies for non-randomized studies.

Meta-analyses of proportions were performed to estimate the bowel preparation adequacy and adenoma/polyp detection rates with shorter versus longer intervals between bowel preparation and colonoscopy. In these meta-analyses, binomial data were used, and the 95% CIs of the estimates were calculated by using score statistics. These meta-analyses incorporated Freeman–Tuckey arcsine transformation for variance stabilization.

Odds ratios depicting the association between the bowel preparation quality and the interval between bowel preparation and colonoscopy were pooled under the random-effects model to achieve an overall point estimate by using the DerSimon–Laird method.

Statistical analyses were performed with Stata software (Stata Corporation, College Station, Tex, USA). All analyses were based on previously published studies; therefore, no ethical approval and informed consent are required.

## Results

**Figure 1.**
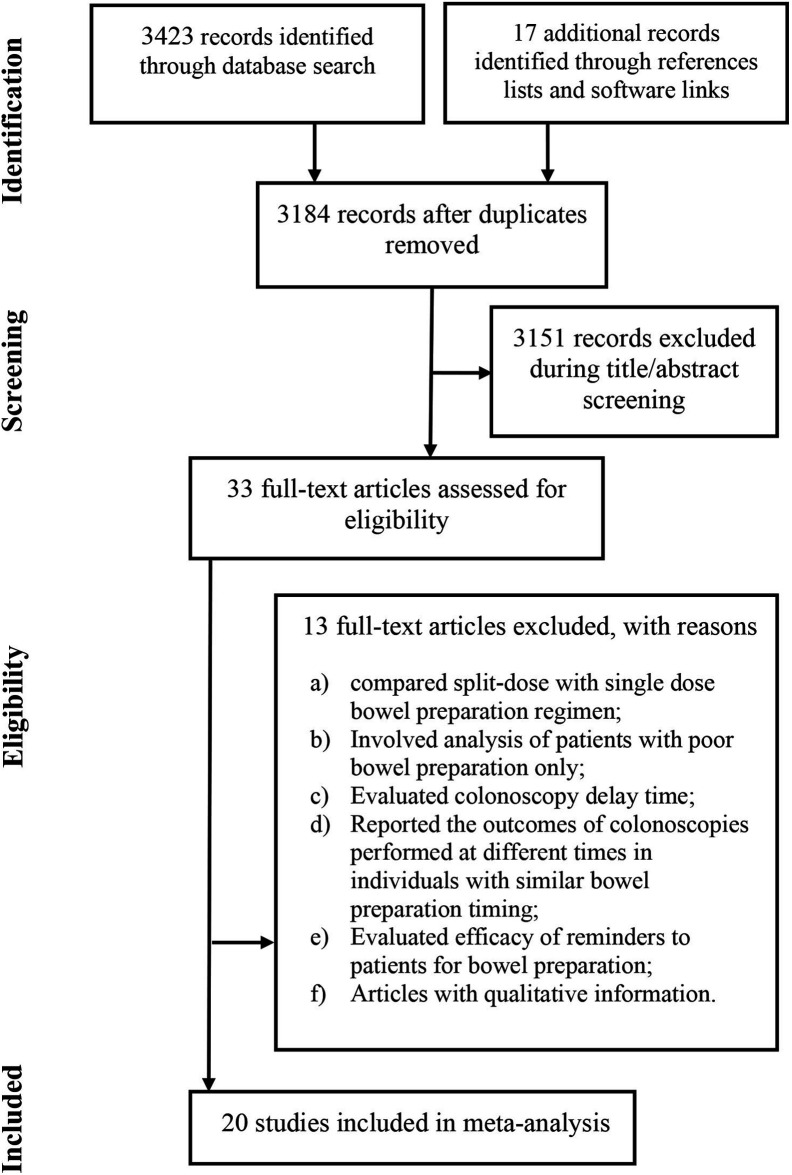
A flowchart of study screening and selection process.

Twenty studies^16-35^ were included (Figure 1). These studies recruited 10 341 individuals for colonoscopy. Of these, 10 were randomized controlled trials, 6 were non-randomized prospective studies, and 4 were retrospective studies. Important characteristics of the included studies are presented in Supplementary Table 1. The quality of the included studies was moderate in general. Many of the randomized studies had high or unclear bias for blinding (Supplementary Table 2). In non-randomized studies, the main constraints were the lack of sample size justification or variance data and no use of blinding of assessment (Supplementary Table 3).

Indications for colonoscopy included diagnosis, treatment, screening, and surveillance. Of these, family history of CRC, neoplasia follow-up, polyp suspicion, hematochezia, anemia, abdominal pain, bowel habit change, diarrhea, colitis, constipation, and significant weight loss were reported by 1 or more studies (Supplementary Table 4).

**Figure 2.**
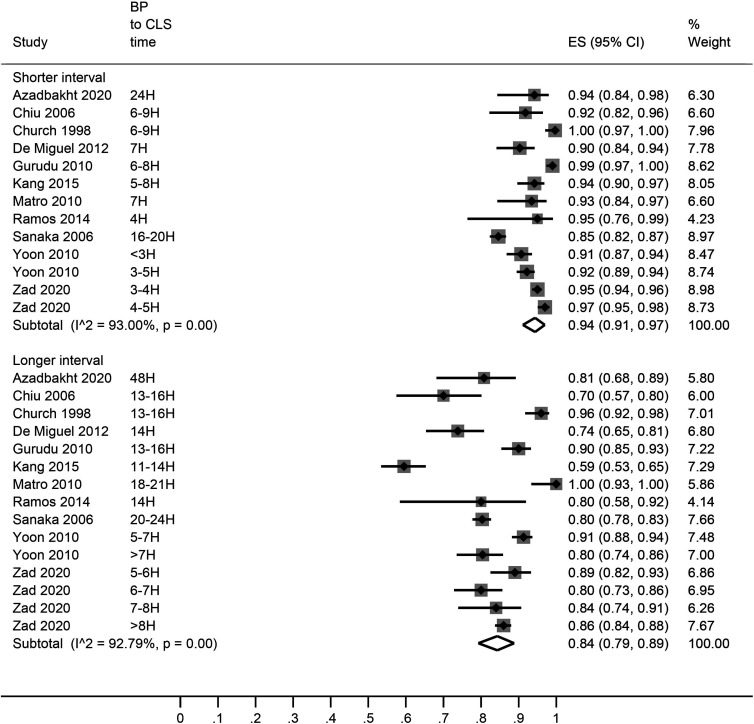
A forest graph showing the pooled estimates of bowel preparation adequacy rates with author-defined shorter and longer bowel preparation to colonoscopy time intervals. ES, effect size.

**Figure 3.**
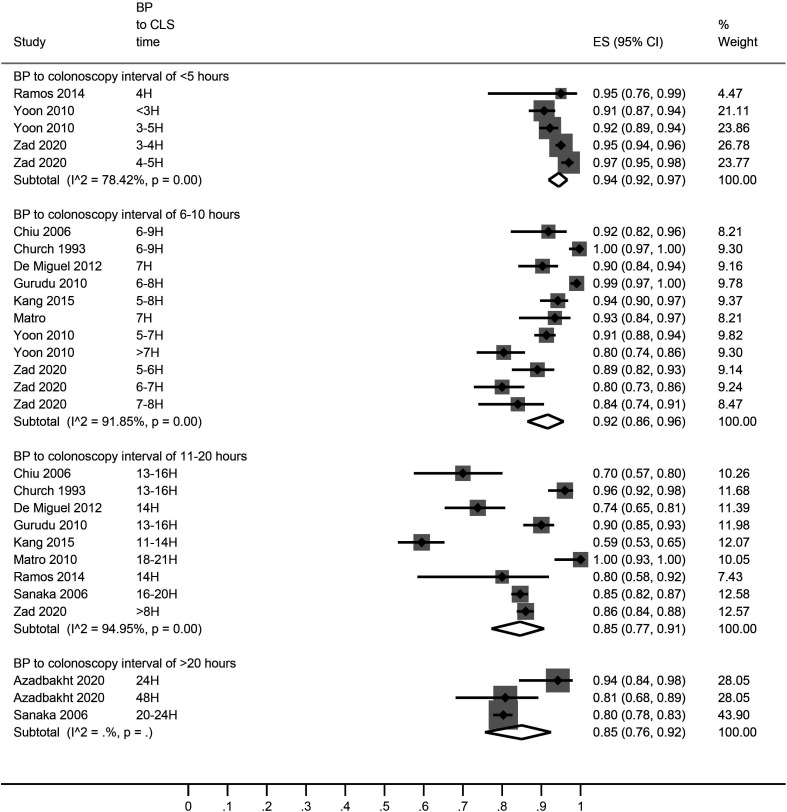
A forest graph showing the pooled estimates of bowel preparation adequacy rates with different bowel preparation to colonoscopy time intervals. BP, bowel preparation; ES, effect size; H, hours.

A pooled analysis of the bowel preparation adequacy rates showed that shorter bowel preparation to colonoscopy time led to better bowel preparation quality (adequacy rate 94% [95% CI: 91, 97]) in comparison with longer interval between bowel preparation and colonoscopy (adequacy rate 84% [95% CI: 79, 89]) (Figure 2). In a subgroup analysis, <5 hours, 6-10 hours, 11-20 hours, and >20 hours intervals were associated with 94% [95% CI: 92, 97], 92% [95% CI: 86, 96], 85% [95% CI: 77, 91], and 85% [95% CI: 76, 92] bowel preparation adequacy rates, respectively (Figure 3). 

**Figure 4.**
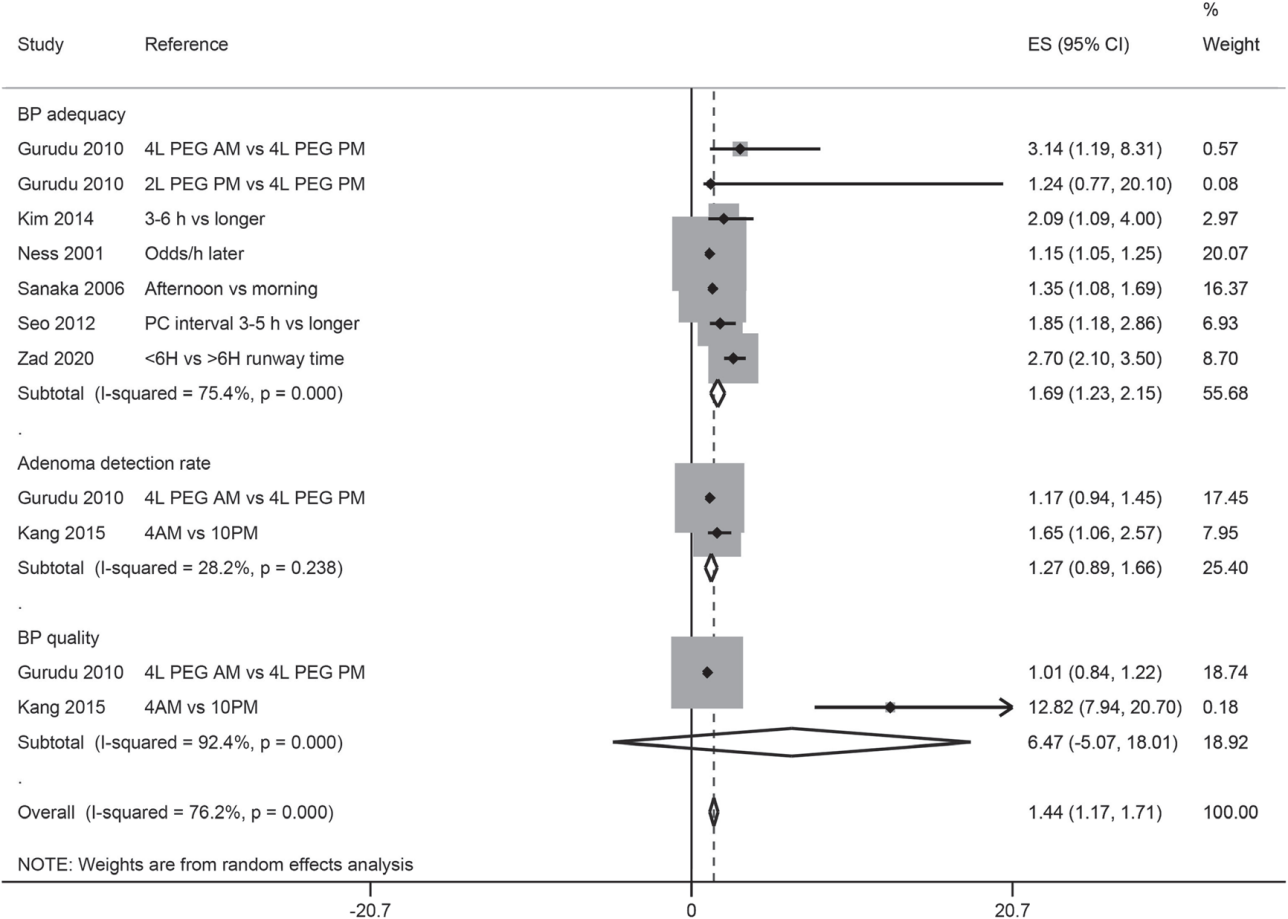
A forest graph showing the pooled estimate of the odds ratios depicting an association between bowel preparation quality and the time interval between bowel preparation and colonoscopy. BP, bowel preparation; ES, effect size; PEG, polyethylene glycol; PC, preparation-to-colonoscopy.

A pooled analysis of the odds ratios reported by the included studies showed that the bowel preparations adequacy was significantly better in shorter bowel preparation to colonoscopy time group in comparison with longer interval group (odds ratio 1.69 [95% CI: 1.23, 2.15]) (Figure 4). Bowel preparation quality scores reported by the included studies are given in Supplementary Table 5.

**Figure 5.**
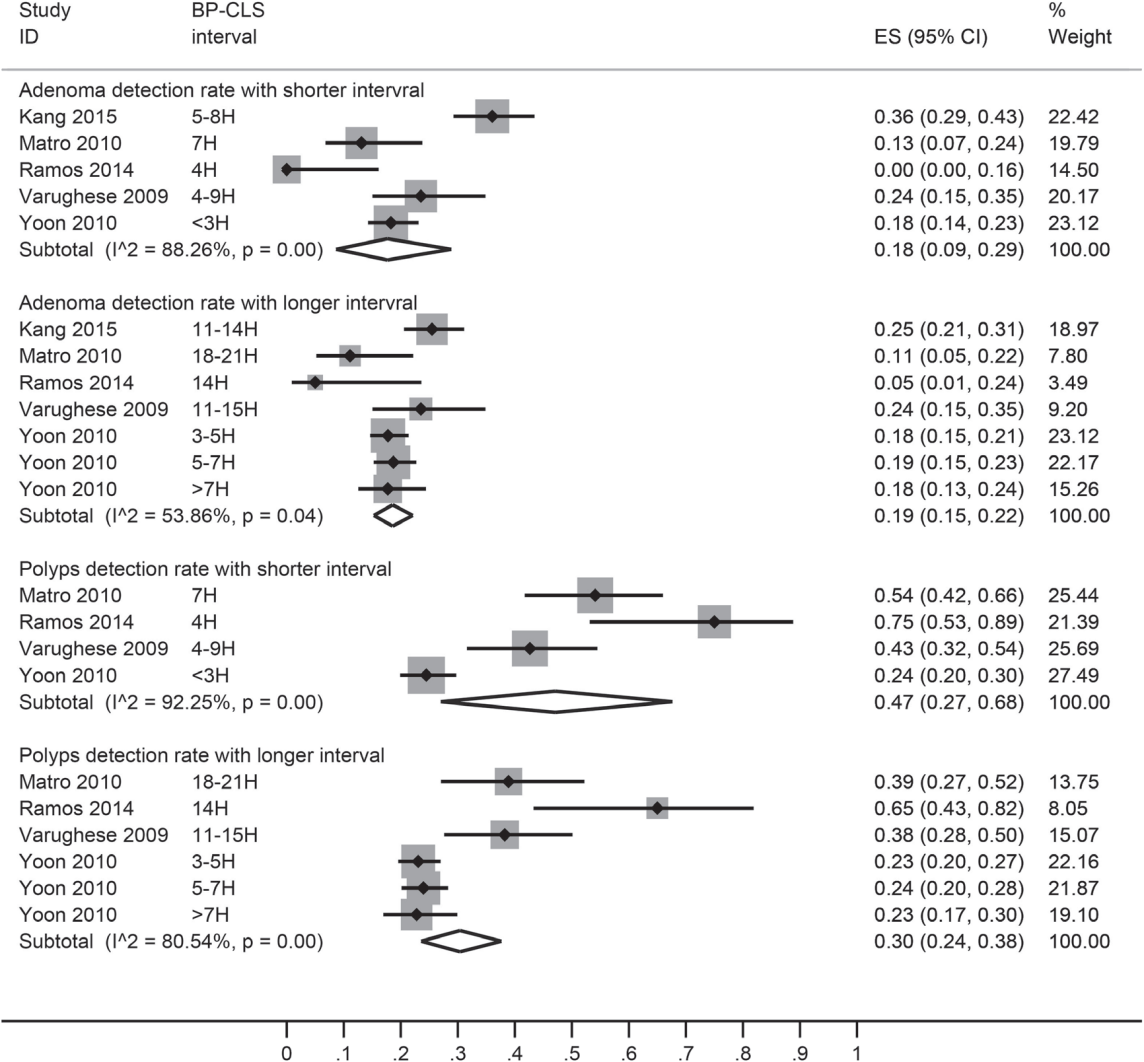
A forest graph showing the pooled estimates of adenoma/polyp detection rates with shorter and longer bowel preparation to colonoscopy intervals. ES, effect size; H, hours.

There was no significant difference in the adenoma detection rate between shorter (18% [95% CI: 9, 29]) and longer (19% [95% CI: 15, 22]) bowel preparation to colonoscopy interval groups (Figure 5). Polyp detection rate was higher in the shorter (47% [95% CI: 27, 68]) than in the longer (30% [95% CI: 24, 38]) bowel preparation to colonoscopy interval group.

## Discussion

The present study has found that a shorter interval between bowel preparation and colonoscopy yields better bowel preparation quality as the bowel preparation adequacy rate was higher with shorter interval in comparison with longer interval. A pooled analysis of the odds ratios reported by the individual studies also endorsed these outcomes. Moreover, a linear trend of declining adequacy rate of bowel preparation was observed from shorter to longer intervals. Many included studies also reported a time-dependent decrease in bowel preparation quality from shorter to longer interval between bowel preparation and colonoscopy.

Bucci et al^15^ performed a meta-analysis of randomized controlled trials comparing split-dose with same-day bowel cleansing regimens and found both regimens to be similar in efficacy. They suggested that a shorter interval between bowel preparation and colonoscopy can improve the efficacy of bowel preparation quality during colonoscopy.^15^ According to the recommendations of the European Society for Gastrointestinal Endoscopy, the last dose of bowel preparation should be given within 5 hours of colonoscopy start and there should not be any ingestion in the last 2 hours before colonoscopy start.^13^ Our results support these recommendations.

In the present study, we have found much variability in determining shorter and longer bowel preparation to colonoscopy intervals. The range of definitions used by different authors for shorter intervals was <3-24 hours and for longer intervals, it was 4-48 hours. Although such an inter-study variability can question the reliability of the main outcomes of this meta-analysis, we have collated evidence from several lines to conclude that shorter time between bowel preparation and colonoscopy leads to better outcomes. These lines included (a) there was a time-dependent decrease in adequacy rates from shorter to longer intervals in a subgroup analysis, (b) a pooled analysis of odds ratios also showed that bowel preparation quality was better with shorter duration, and (c) in general, bowel preparation scale scores were better in shorter interval groups than in longer interval groups.

The diagnostic accuracy of colonoscopy is affected by bowel preparation. Inadequate bowel cleansing can not only reduce the efficiency of examination but can also increase the chances of complications and time utilization.^17^ The outcomes that a shorter bowel preparation to colonoscopy interval yields better bowel preparation quality may have an association with the gut functionality. It is known that colonic debris consists of fecal material and gut secretions. Purgatives are usually osmotically balanced and non-absorbable agents are used to induce stools for bowel cleansing. However, with the passage of time, gut secretions keep on accumulating and can make hurdles in endoscopic visualizations.^22^ Our results suggest that the time of day when colonoscopy is performed may not affect the quality of bowel preparation but the time interval between bowel preparation and colonoscopy does. Although some studies have suggested that evening time colonoscopies are associated with lower quality of bowel preparation,^29,36^ the time interval between bowel preparation and colonoscopy could be longer in these studies.^29^

A study of over 200 outpatients who received sodium phosphate for bowel preparation found that the quality of bowel preparation was negatively correlated with the interval between the last dose and colonoscopy.^37^ Siddiqui et al^32^ who reported adequacy rates at several time points, found the highest bowel preparation adequacy rate with 8 hours interval between bowel preparation and colonoscopy. Thus, although these outcomes favor a shorter interval between bowel preparation and colonoscopy, optimal time is not fully clear as there was a wide range of definitions of shorter intervals among the included studies. Seo et al^30^ suggested that an optimal bowel preparation to colonoscopy interval should be 3-5 hours with split-dose purgatives. Although in one of the subgroup analyses we have found the highest bowel preparation adequacy rate with an interval of <5 hours, adequacy rates of over 90% were also observed with relatively longer durations by some studies (Figure 3).

In the present study, we have found that there was no significant difference between shorter and longer bowel preparation to colonoscopy intervals in adenoma detection rate. Among the individual studies, only Kang et al^23^ found a significantly lower adenoma detection rate in the longer interval group in comparison with the shorter interval group. Parra-Blanco et al^27^ found a statistically significantly higher flat lesion detection rate in shorter bowel preparation to colonoscopy interval. Chiu et al^17^ found a significantly lower total lesion detection rate in the longer interval group (2.78 ± 0.29 vs 1.9 ± 0.27; *P* = .026).

The polyp detection rate was higher in the shorter interval group in the present study. Other studies have also found a positive association between bowel preparation quality and polyp detection rate. A study of more than 600 patients in which telephonic reminders were given in the intervention group that was compared to a control group in which patients were not given reminders found better bowel preparation quality in the intervention group which was also associated with higher polyp detection rate.^38^ On the other hand, a study in which patients were given a reinforced education by telephone and short message services found no difference in bowel preparation quality between the intervention and control groups, and this was also associated with no differences in polyp detection rates.^39^

Further studies are required to determine optimal time interval between bowel preparation and colonoscopy. Moreover, standardization of adequacy rates, bowel preparation schedule definition, patient’s health literacy, and analyses with risk moderation will be needed to ensure reliable outcomes. Several risk factors for bowel preparation inadequacy are identified. These include age >60 years, diabetes, history of appendectomy, history of colorectal resection, history of cirrhosis, history of hysterectomy, history of stroke, a history of dementia, interpreter requirement, health insurance, endoscopist, single status, multiple prescription medications, reported failure to follow preparation instruction, inpatient status, constipation as an indication for colonoscopy, tricyclic antidepressants, and male gender.^26,40-42^ Thus, patient group identification or individualized bowel preparation schedule can be more useful for the attainment of required bowel preparation quality for colonoscopy. Unlike other factors affecting the quality of bowel preparation, the time interval between bowel preparation and colonoscopy is a modifiable factor that should be worked out to determine the optimal time that would improve diagnostic efficiency and reduce health care costs.

## Conclusion

Shorter bowel preparation to colonoscopy time interval is found to be associated with better bowel preparation quality. However, due to the use of a wide range of definitions of shorter and longer intervals by the individual studies, an optimal time interval between bowel preparation and colonoscopy could not be determined. The adenoma detection rate was not different among the shorter and longer interval groups. However, the polyp detection rate was higher with shorter bowel preparation to colonoscopy interval. 

## Figures and Tables

**Supplementary Table 1.** Important Characteristics of the Included Studies

Study ID	**n**	**Design**	**Age (Years)**	**Females (%)**	**Body Mass Index (kg/m ^2^ )**	**Constipation History (%)**	**Diabetes (%)**	**Surgery History (%)**	**BP to Colonoscopy Time (hours)**	
									**Shorter**	**Longer**
**Azadbakht 2020**	104	RCT							24	48
**Chiu 2006**	121	RCT	57 ± 10	23	24.7 ± 0.45			16	6-9	13-16
**Church 1993**	317	RCT						18		
**De Miguel 2012**	256	RCT	62 ± 5.2	36		23	3		6.5	14
**Eun 2011**	300	PROSP	52 ± 13	49	23.5 ± 3.2	11		11	<5	<6/<7/<8/<9/>10
**Gupta 2007**	201	RCT							3	16-18
**Gurudu 2010 **	602	RETRO	61 ± 13	48					6-8	13-16
**Kang 2015**	431	PROSP	48 ± 10	39	39% >27				5-8	11-14
**Kim 2014**	165	PROSP		35	24.2 ± 3.1	17	10	12	3-4	4-5/5-6/6-7/7-8/>8
**Matro 2010**	115	RCT	52 ± 12	55			8	37		
**Ness 2001**	649	RETRO	56 ± 14	54	27.3 ± 6.8	5	14	35	7 H	18-21 H
**Parra-Blanco 2006**	88	RCT	56 ± 16	43		21			6-1	11-17
**Ramos 2014**	40	RCT	62 ± 15	70					4	14
**Sanaka 2006**	2083	RETRO	55 ± 13	62		22		16	16-20	20-24
**Seo 2012**	366	PROS		52	23 ± 2.6	20	7	25	3-4	4-5/5-6/6-7/7-8/>8
**Shah 2014**	200	RCT	52 + 16						4-6 H	14-16 H
**Siddiqui 2009**	100	PROS		4		23			8	10/12/14/16/18/20
**Varughese 2009**	136	RCT	52 ± 11	48	28.5 ± 5.65				4-9	11-15
**Yoon 2010**	780	PROS	52 ± 11	39	23.7 ± 2.9				<	<5/<7/>7
**Zad 2020**	3295	RETRO	72% >50	45	64% OW/Ob		13		16-18	18-20

BP, bowel preparation; H, hours; OW/ob, overweight/obese.

**Supplementary Table 2.** Quality Assessment of the Randomized Controlled Trials

Study	**Other Bias**	**Selective Reporting**	**Incomplete Outcome Data**	**Blinding of Outcome Assessment**	**Blinding of Participants /Personnel**	**Allocation Concealment**	**Random Sequence Generator**
Azadbakht 2020	U	L	H	H	H	NA	L
Chiu 2006	U	L	H	L	L	NA	L
Church 1993	U	H	H	L	L	NA	L
De Miguel 2012	U	L	L	U	U	NA	L
Gupta 2007	U	H	H	L	L	NA	L
Matro 2010	U	L	L	U	L	NA	L
Parra-Blanco 2006	U	L	L	U	U	NA	L
Ramos 2014	U	H	H	U	L	NA	L
Shah 2014	U	H	H	U	L	NA	L
Varughese 2009	U	L	L	L	L	NA	L

H, high risk; L, low risk; U, unclear risk; NA, not applicable.

**Supplementary Table 3.** Quality Assessment of Included Studies of Non-randomized Studies

Criteria	Eun 2011	Gurudu 2010	Kang 2015	Kim 2014	Ness 2001	Sanaka 2006	Seo 2012	Siddiqui 2009	Yoon 2010	Zad 2020
1. Was the research question or objective in this paper clearly stated?	Y	Y	Y	Y	Y	Y	Y	Y	Y	Y
2. Was the study population clearly specified and defined?	Y	Y	Y	Y	Y	Y	Y	Y	Y	Y
3. Was the participation rate of eligible persons at least 50%?	Y	Y	Y	Y	Y	Y	Y	Y	Y	Y
4a. Were all the subjects selected or recruited from the same or similar populations (including the same time period)?	Y	Y	Y	Y	Y	Y	Y	Y	Y	Y
4b. Were inclusion and exclusion criteria for being in the study prespecified and applied uniformly to all participants?	Y	N	Y	Y	N	N	Y	Y	N	N
5. Was a sample size justification, power description, or variance and effect estimates provided?	N	N	Y	N	N	N	Y	N	N	N
6. For the analyses in this paper, were the exposure(s) of interest measured prior to the outcome(s) being measured?	Y	Y	Y	Y	Y	Y	Y	Y	Y	Y
7. Was the timeframe sufficient so that one could reasonably expect to see an association between exposure and outcome if it existed?	Y	Y	Y	Y	Y	Y	Y	Y	Y	Y
8. For exposures that can vary in amount or level, did the study examine different levels of the exposure as related to the outcome (e.g., categories of exposure, or exposure measured as continuous variable)?	Y	N	N	Y	N	N	Y	Y	Y	Y
9. Were the exposure measures (independent variables) clearly defined, valid, reliable, and implemented consistently across all study participants?	NR	Y	Y	NR	N	N	NR	NR	NR	Y
10. Was the exposure(s) assessed more than once over time?	N	N	N	N	N	N	N	N	N	N
11. Were the outcome measures (dependent variables) clearly defined, valid, reliable, and implemented consistently across all study participants?	Y	Y	Y	Y	Y	Y	Y	Y	Y	Y
12. Were the outcome assessors blinded to the exposure status of participants?	N	N	NR	NR	N	N	Y	NR	NR	N
13. Was loss to follow-up after baseline 20% or less?	NA	NA	NA	NA	NA	NA	NA	NA	NA	NA
14. Were key potential confounding variables measured and adjusted statistically for their impact on the relationship between exposure(s) and outcome(s)?	NR	Y	Y	NR	N	N	NR	NR	NR	Y

Y, yes; N, no; NR, not reported; NA, not applicable.

**Supplementary Table 4.** Indications for Colonoscopy Reported by the Included Studies

Study	**Indications**
Church 1993	Surveillance for neoplasia (50%), poly suspicion (11%), screening for family history of CRC (14%), symptoms (14%)
Eun 2011	Asymptomatic screening (25%), screening for family history of CRC (2%), surveillance for neoplasia (5.7%), polyp suspicion (6%), rectal bleeding 12%), anemia (3%), abdominal pain (30%), bowel habit change (13%)
Grudu 2010	Screening/surveillance (61%), Anemia/bleeding (11%), abdominal pain (4%), diarrhea (8%), constipation (2%), colitis (3)
Matro 2010	Screening (51%), surveillance (17.5%), symptomatic (31.5%)
Ramos 2014	Incomplete colonoscopy (80%), Colonoscopy contraindication or rejection (20%)
Seo 2012	Screening (40%), surveillance (17%), positive stool occult blood (2), rectal bleeding (6%), abdominal pain/bloating (25%), anemia (1%), bowel habit change (3%), diarrhea (2%), constipation (4%), significant weight loss (1%)
Sorser 2015	Rectal bleeding (66%), diarrhea (31%), abdominal pain (30%), Crohn’s disease (6%)
Varughese 2009	Screening/surveillance (53%), diagnostics (47%)
Zad 2020	Bowel habit change (14%), screening/surveillance (32%, abdominal pain (7%), gut bleeding / anemia (42%)

**Supplementary Table 5.** Bowel Preparation Quality Scale Scores

Study	nS	nL	Bowel Preparation Quality Scale	Comparators	Shorter Interval	Longer Interval
De Miguel 2012	134	122	Boston BPS	<7 H vs 14 H	7.94 ± 1.43	7.48 ± 1.63
Eun 2011	23	67	Ottawa BPS	<5 H vs 6 H	3.5	3.8
Eun 2011	23	86	Ottawa BPS	<5 H vs 7 H	3.5	3.4
Eun 2011	23	69	Ottawa BPS	<5 H vs 8 H	3.5	3.8
Eun 2011	23	20	Ottawa BPS	<5 H vs 9 H	3.5	4.8
Eun 2011	23	28	Ottawa BPS	<5 H vs >10 H	3.5	5
Gupta 2007	102	99	Ottawa BPS	3-6 H vs 16-18 H	4.7±2.8	4.7±2.8
Kim 2014	13	27	Ottawa BPS	3-4 H vs >4-5 H	6.67	6.93
Kim 2014	13	37	Ottawa BPS	3-4 H vs 5-6 H	6.67	6.35
Kim 2014	13	33	Ottawa BPS	3-4 H vs 6-7 H	6.67	7.03
Kim 2014	13	23	Ottawa BPS	3-4 H vs 7-8 H	6.67	7.52
Kim 2014	13	25	Ottawa BPS	3-4 H vs >8 H	6.67	9.04
Parra-Blanco 2006	43	45	Self-designed^*^	Cleansing quality >4	78.6 ± 0.2	26.7 ± 0.02
Seo 2012	58	51	Ottawa BPS	3-4 H vs 4-5 H	4.25	4.7
Seo 2012	58	35	Ottawa BPS	3-4 H vs 5-6 H	4.25	5.11
Seo 2012	58	53	Ottawa BPS	3-4 H vs 6-7 H	4.25	4.86
Seo 2012	58	62	Ottawa BPS	3-4 H vs 7-8 H	4.25	5.2
Seo 2012	58	39	Ottawa BPS	3-4 H vs >8 H	4.25	5.92
Shah 2014	203	97	Ottawa BPS	4-6 H vs >6 H	6.02 ± 1.34	5.52 ± 1.23
Siddiqui 2009			Self-designed^#^	8 H vs 10 H	0.8	1.45
Siddiqui 2009			Self-designed	8 H vs 12 H	0.8	1.6
Siddiqui 2009			Self-designed	8 H vs 14 H	0.8	1.5
Siddiqui 2009			Self-designed	8 H vs 16 H	0.8	1.7
Siddiqui 2009			Self-designed	8 H vs 18 H	0.8	1.7
Siddiqui 2009			Self-designed	8 H vs 20 H	0.8	2.2

BPS, bowel preparation quality scale; H, hour/s; nS/nL, sample sizes of shorter/longer interval groups.

^*^Scale: 5 excellent (no material or liquid material covering < 10% of the mucosal surface in each location), 4 good (liquid material or mucus covering >10% of the mucosal surface), 3 acceptable (small particles easy to suction), 2 fair (solid material impossible to suction, covering < 10% of the mucosal surface), 1 poor (solid material covering > 10% of the mucosal surface). Global quality was calculated as the arithmetic mean of the quality in the different locations.

^#^Scale: 4, unsatisfactory; 3, poor; 2, fair; 1, good; a nd 0, excellent.
